# Wnt Signaling and Its Impact on Mitochondrial and Cell Cycle Dynamics in Pluripotent Stem Cells

**DOI:** 10.3390/genes9020109

**Published:** 2018-02-19

**Authors:** Megan L. Rasmussen, Natalya A. Ortolano, Alejandra I. Romero-Morales, Vivian Gama

**Affiliations:** 1 Department of Cell and Developmental Biology; Vanderbilt University, Nashville, TN 37232, USA; megan.merolla@vanderbilt.edu (M.L.R.); natalya.a.ortolano@vanderbilt.edu (N.A.O.); a.romeromorales@vanderbilt.edu (A.I.R.-M.); 2 Vanderbilt Center for Stem Cell Biology; Vanderbilt University, Nashville, TN 37232, USA; 3 Vanderbilt Ingram Cancer Center; Vanderbilt University, Nashville, TN 37232, USA

**Keywords:** stem cells, pluripotency, mitochondria, cell cycle, apoptosis, Wnt

## Abstract

The core transcriptional network regulating stem cell self-renewal and pluripotency remains an intense area of research. Increasing evidence indicates that modified regulation of basic cellular processes such as mitochondrial dynamics, apoptosis, and cell cycle are also essential for pluripotent stem cell identity and fate decisions. Here, we review evidence for Wnt regulation of pluripotency and self-renewal, and its connections to emerging features of pluripotent stem cells, including (1) increased mitochondrial fragmentation, (2) increased sensitivity to cell death, and (3) shortened cell cycle. We provide a general overview of the stem cell–specific mechanisms involved in the maintenance of these uncharacterized hallmarks of pluripotency and highlight potential links to the Wnt signaling pathway. Given the physiological importance of stem cells and their enormous potential for regenerative medicine, understanding fundamental mechanisms mediating the crosstalk between Wnt, organelle-dynamics, apoptosis, and cell cycle will be crucial to gain insight into the regulation of stemness.

## 1. Introduction

Embryonic stem cells (ESCs) are derived from the inner cell mass (ICM) of the blastocyst [1,2,3]. Inner cell mass derived ESCs model the earliest stages of mammalian development, providing a useful tool for in vitro studies, yet the molecular mechanisms governing key signaling pathways and other non-transcriptional regulators of stemness are poorly characterized. ESCs are both self-renewing and pluripotent. The abilities to divide indefinitely and to generate any cell in the body have launched an entire field of research dedicated to manipulating growth factors and signaling pathways to mimic embryonic development in the culture dish. The Wnt signaling cascade is an evolutionarily conserved regulator of organismal development that was identified early on as a crucial factor for the maintenance of self-renewing and pluripotent capacities. Wnt is also a key driver of tissue morphogenesis and architecture. In this review, we will focus our discussion on the role of Wnt in mitochondrial dynamics, apoptosis, and cell cycle in the context of pluripotency. Covering other developmental aspects of Wnt signaling is beyond our scope. Excellent reviews on these topics can be found elsewhere [4,5,6,7].

**Figure 1:**
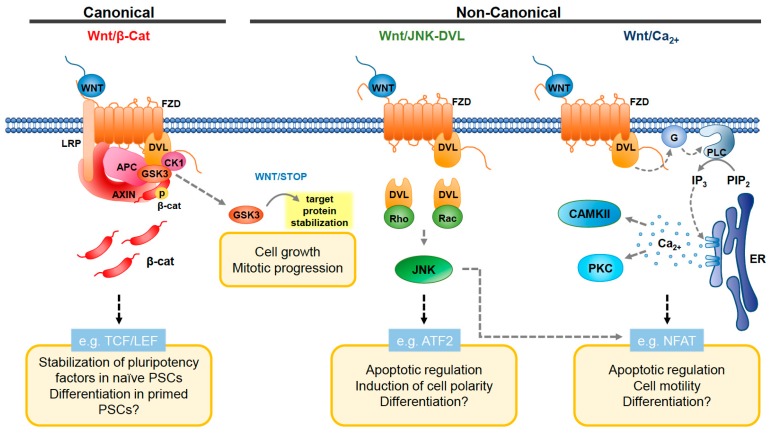
Canonical and non-canonical Wnt pathways and their effects in pluripotent stem cells (PSCs). Canonical Wnt/β-catenin pathway activation (**left**) starts with WNT binding Frizzled (FZD) receptor and the co-receptor LRP5/6, which induces the recruitment of Dishevelled (DVL)and inhibits the β-catenin destruction complex formed by AXIN, APC, GSK3, and CK1. This inhibition causes the accumulation of β-catenin, which is no longer phosphorylated by the destruction complex. β-catenin then translocates to the nucleus where it activates transcription of Wnt target genes. A divergent pathway involving GSK3 participates in the stabilization of proteins important in mitotic progression (Wnt/Stabilization of proteins (STOP) pathway). In the non-canonical Wnt/DVL-JNK pathway (**middle**), DVL activation by Wnt induces the activation of Rho and Rac small guanosine triphosphatases (GTPases). Activation of these proteins signals through JNK. In the non-canonical Wnt/Ca^2+^ pathway (**right**), the binding of WNT to FZD activates the heterotrimeric G-proteins. These signal through phospholipase C (PLC) and inositol 1,4,5-triphosphate (IP_3_) to induce the release of intracellular Ca^2+^ and the activation of Protein Kinase C (PKC) and Ca^2+^/calmodulin-dependent protein kinase II (CaMKII). NFAT: Nuclear factor of activated T-cells; APC: adenomatosis polyposis coli; ER: Endoplasmic reticulum; IP_3_: Inositol 1,4,5-triphosphate; PIP_2_: Phosphatidylinositol 4,5-bisphosphate

Wnt ligands are secreted signaling proteins, often acting as morphogens, that can activate several signaling pathways: (1) the Wnt/β-catenin pathway, (2) the planar cell polarity pathway, and (3) the Wnt/Calcium (Ca^2+^) pathway (Figure 1). The Wnt receptor, Frizzled (FZD), is the starting point of each of these pathways, leading to recruitment of Dishevelled (DVL), which then relays the signal to downstream players of each pathway. The canonical Wnt/β-catenin pathway is specifically mediated by WNT binding to FZD and its co-factor, LDL-receptor-related protein (LRP). In the absence of Wnt ligand, glycogen synthase kinase 3 (GSK3) represses the pathway by targeting β-catenin for destruction by the degradation complex, formed in combination with AXIN, Casein kinase 1 (CK1), and adenomatosis polyposis coli (APC) [8]. WNT binding to FZD signals for dissociation of the degradation complex, allowing for the stabilization of β-catenin and its translocation to the nucleus. Nuclear β-catenin then interacts with and activates transcription factors (i.e., T-cell factor/lymphoid enhancer factor (TCF/LEF)), thereby promoting Wnt target gene expression [9]. Interestingly, despite the inactivity of the transcriptional machinery during mitosis, high Wnt activity is maintained [10,11]. Recent studies determined that this peak in Wnt signaling regulates not only β-catenin stability, but also the stability of proteins unrelated to transcription. In fact, over 20% of the proteome contains GSK3 phospho-degrons, suggesting that GSK3 targets are regulated by Wnt signaling [12]. These GSK3-regulated proteins are stabilized during mitosis, ultimately increasing cell size and supporting cell division [11]. This β-catenin-independent function of Wnt signaling is known as Wnt-dependent stabilization of proteins (Wnt/STOP). Wnt/STOP seems to be the predominant Wnt pathway in many cancer cells, where it may play a key role in promoting cell growth [11].

Other non-canonical Wnt signaling is mediated either by Ca^2+^ or the c-Jun NH (2)-terminal kinase (JNK) pathway. In the Wnt/Ca^2+^ pathway, Wnt ligands bind DVL and FZD to activate phospholipase C (PLC), which converts phosphatidylinositol 4,5-bisphosphate (PIP_2_) into inositol 1,4,5-triphosphate (IP_3_). Inositol 1,4,5-triphosphate diffuses into the cytosol and interacts with Ca^2+^ channels at the Endoplasmic reticulum (ER) membrane to facilitate the release of Ca^2+^ ions. As a result, several downstream targets are activated, including protein kinase C-cell division control protein 42 (PKC-CDC42) [13,14], Ca^2+^/calmodulin-dependent protein kinase II (CAMKII) [15], transforming growth factor β (TGFβ) activated kinase (TAK1) and Nemo-like kinase (NLK) [16]. In the Wnt/DVL-JNK pathway, downstream targets of DVL, Rho, and Rac work to activate the JNK signaling. This signaling network is referred to as the planar cell polarity (PCP) pathway, since it is involved in cellular polarity, cytoskeleton assembly, and migration of cells during embryonic development [9]. Interestingly, there appears to be cross talk between the PCP pathway and the β-catenin pathway. LRP6, the Wnt co-receptor, is required for canonical Wnt signaling. However, its downregulation results in the activation of the PCP pathway, disrupting gastrulation and cell migration in the developing *Xenopus* embryo [17]. This observation suggests that LRP6 plays a critical role in the developmental switch from PCP to Wnt/β-catenin pathways.

While many developmental signaling programs have been shown to have opposing roles in mouse and human ESCs (e.g., bone morphogenic protein (BMP), leukemia inhibitory factor (LIF)), Wnt is one of the few pathways that are fundamental for self-renewal in both systems. For example, WNT3 mutations in mice prevent the embryo from completing gastrulation [18], and human Wnt dysregulation results in a myriad of developmental disorders, such as abnormalities in bone density, neural tube formation, and retinal development [4,19]. Most tissues have prominent stem cell niches that are crucial during development. Thus, discoveries on how the Wnt pathway is modulated in these niches during organismal development have greatly impacted the stem cell field, leading to new advances and applications.

Wnt-mediated signaling plays reciprocal roles in pluripotent stem cells (PSCs: referring to both ESCs and induced pluripotent stem cells (iPSCs)), regulating both self-renewal and differentiation into mature cell types. It is known to promote and maintain pluripotency [20], as well as to stimulate somatic cell reprogramming [21]. The Wnt receptor, frizzled family receptor 7 (FZD7), has been shown to be essential for preventing spontaneous differentiation of human ESCs (hESCs), and therefore a continuous signaling loop is required for pluripotent maintenance [22]. Wnt-conditioned medium is able to replace retroviral c-MYC in reprogramming protocols, with the resulting colonies appearing identical to iPSCs and ESCs [21]. Wnt pathway manipulation is particularly important in the preservation of self-renewal capacity in vitro [23]. Specifically, this regulation has been attributed to stabilization of NANOG levels by Wnt activation, which leads to Estrogen-related receptor b (ESRRB) activation. Estrogen-related receptor b has been shown to be a target of the GSK3/TCF3 axis in the canonical Wnt pathway [24,25]. Sato et al., showed that activation of Wnt signaling through the inhibition of GSK3 using the pharmacological inhibitor 6-bromoindirubin-3’-oxime (BIO) promoted pluripotent maintenance of both human and mouse ESCs [20]. Aside from self-renewal, Wnt pathways have been the focus of manipulation in many differentiation protocols to the three germ lineages [9].

Wnt-mediated control of intrinsic stem cell characteristics is particularly variable between the two pluripotent states: primed and naïve [26]. Two distinct pluripotent states are observed in stem cells from mice: the ground or naïve state, exemplified by mouse embryonic stem cells (mESCs) [2], and the primed pluripotent state represented by mouse epiblast stem cells (mEpiSCs) [27,28]. Human embryonic stem cells and human induced pluripotent stem cells (hiPSCs) identify more closely with mEpiSCs than mESCs [28]. The role of Wnt in the induction and maintenance of these states is controversial. Small molecule inhibition of Wnt pathway components can replace fetal bovine serum in hESCs, which are not LIF dependent, reverting them back to a naïve state [29]. Cells cultured in this media (termed 3-inhibitor or 3i media) were shown to have a similar epigenetic landscape to pre-implantation blastocysts [30]. It was later discovered that the combination of PD0325901 (a mitogen-activated protein kinase/extracellular-signal-regulated kinase (MAPK/ERK) inhibitor) with CHIR99021 (a GSK3 inhibitor), collectively termed 2-inhibitor or 2i media, was efficient for the culture of both ESCs and iPSCs [29,31,32]. Likewise, Wnt stimulation in naïve mESCs maintained in 2i/LIF media made them resistant to differentiation. However, the same treatment in mEpiSCs maintained in normal serum/LIF media stimulated differentiation [20]. In contrast, another study utilizing a β-catenin-deficient mESC line reported no requirement for canonical Wnt signaling in maintaining self-renewal [33]. Instead, these cells had impaired germ layer differentiation caused by β-catenin-dependent cell-adhesion dysfunction. In hESCs, however, Wnt activation promoted their differentiation [34]. These conflicting data indicate that the role of Wnt signaling in PSCs is diverse and context-dependent. Therefore, systematic assessment into culture conditions, mechanism of Wnt activation/inhibition, and pluripotent state (i.e., naïve or primed) will be needed to uncover the intersections between Wnt pathways and their effect on modulating pluripotency. In addition, the roles of Wnt in other processes associated with pluripotency are unclear. Here, we review several biological features essential for stem cell biology and the known roles for Wnt signaling. These processes include mitochondrial dynamics, intrinsic apoptosis, and cell cycle regulation. We also consider new relationships between Wnt pathway components, stem cell maintenance, and differentiation.

## 2. Wnt Pathway and Mitochondrial Dynamics

The balance between mitochondrial fission (fragmentation) and fusion, known as mitochondrial dynamics, is key to cellular homeostasis. Maintaining this delicate balance allows cells to preserve mitochondrial genome integrity, generate ATP, and control the production of reactive oxygen species (ROS) [35]. The formation of mitochondrial networks in mature cells by increased fusion has been shown to be key for mitochondrial DNA (mtDNA) homogenization and efficient assembly of the electron transport chain (ETC) [36]. On the other hand, mitochondrial fission is necessary during cell division and for segregation of damaged mitochondria for mitophagy [37]. Mitochondrial structural changes have dramatic effects on cellular metabolism [38,39,40] and have been linked to both neurodegenerative and neurodevelopmental diseases such as Parkinson’s, Alzheimer’s, and Charcot-Marie-Tooth disease [41]. Metabolic adaptations triggered by mitochondrial dynamics can influence metabolite levels, redox state and cell cycle, impacting stem cell survival and function [42,43,44,45].

### 2.1. Mechanisms of Mitochondrial Dynamics Machinery

**Figure 2:**
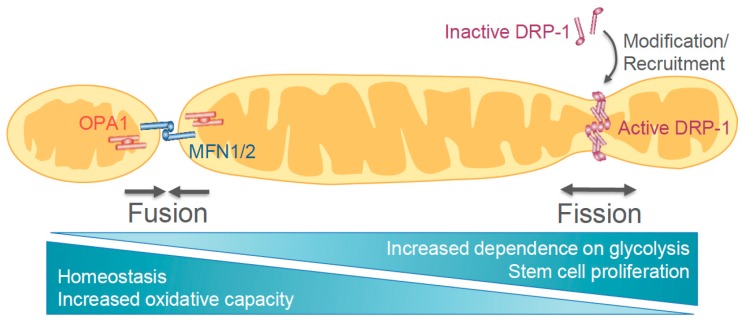
Mitochondrial dynamics are regulated by several large guanosine triphosphatases (GTPases). Mitochondrial fusion is mediated by the activity of mitofusins (MFN) 1 and MFN2 at the outer mitochondrial membrane, while optic atrophy 1 (OPA1) regulates fusion of the inner mitochondrial membrane. Fission occurs through recruitment of active dynamin-related protein 1 (DRP-1) to receptors at the outer mitochondrial membrane by various mechanisms, including phosphorylation, ubiquitination, and sumoylation.

Mitochondrial dynamics machinery is comprised of several dynamin superfamily guanosine triphosphatases (GTPases) that have roles in either fission or fusion of mitochondria (Figure 2). Dynamin-related protein 1 (DRP-1) is required for mitochondrial fission. DRP-1 activation is mediated in part by phosphorylation, ubiquitination, and sumoylation, which allow for increased recruitment to various receptors (e.g., mitochondrial fission protein 1 (FIS1)) [46,47,48]. Once activated, DRP-1 oligomerizes around the organelle and constricts, dividing it into two separate mitochondria [49]. Other GTPases important for mitochondrial dynamics are the mitofusins (MFN), MFN1 and MFN2 [50]. These proteins are anchored in the outer mitochondrial membrane and facilitate fusion by forming homo- or hetero-dimers with MFNs on nearby target mitochondria [49]. After fusion of the outer mitochondrial membrane, the inner membrane fuses through a similar mechanism mediated by optic atrophy 1 (OPA1) [51,52]. OPA1 is localized to the inner mitochondrial membrane with its GTPase domain exposed to the inner membrane space (IMS) [52,53]. OPA1 is increasingly proteolytically cleaved in metabolically stressed cells, rendering it inactive and resulting in reduced cristae integrity and mitochondrial fragmentation [51].

Stem cells have high mitochondrial fragmentation mediated through DRP-1 activity [54]. Phosphorylation of DRP-1 promotes mitochondrial fragmentation, which is followed by an increase in glycolytic activity and conservation of the cell’s proliferative capacity [55]. Mitochondrial fission is essential for efficient reprogramming of fibroblasts into iPSCs. Interestingly, the recruitment of DRP-1 during early phases of reprogramming generates a severe mitochondrial fragmentation phenotype, followed by the restoration of the network into shorter tubules once pluripotent colonies are formed [56]. While it is known that DRP-1-dependent mitochondrial fragmentation is necessary for acquiring pluripotency [55,56,57], the exact post-translational modifications of DRP-1 and the temporal regulation of mitochondrial machinery during reprogramming remain unclear.

Interactions between the Wnt pathway and mitochondrial dynamics modulators have been identified in several tissues. In hippocampal neurons of the central nervous system, the non-canonical WNT5A ligand induces the traffic and retention of gamma-aminobutyric acid receptor A (GABA-A) [58], which results in an increase in cellular Ca^2+^. This spike in Ca^2+^ resulted in increased activation of DRP-1, at least in part, by CAMKII-mediated phosphorylation at serine 616 [59]. The net result is the redistribution of mitochondria to newly formed neural spines in the hippocampus [59]. Also, it has been shown that aberrant mitochondrial fission at the postsynaptic region in neurons could be mediated by WNT5A [60,61]. This fragmentation seems to be promoted by increased concentrations of intracellular and mitochondrial Ca^2+^ levels that promote the activity of DRP-1. The activation of DRP-1 is thought to be mediated by PKC and calcineurin (CaN) [60,61].

In murine muscle cell lines, connections between Wnt and mitochondrial biogenesis or activity were identified by a large-scale RNA interference (RNAi) screen for mitochondrial regulators. Key fundamental components of the canonical Wnt pathway were found to modulate mitochondrial mass and membrane potential. When recombinant WNT3A was incorporated to the growth media, mitochondrial biogenesis increased in a dose-dependent manner, with a concomitant increase in respiratory capacity and mitochondrial volume and length [62]. Intriguingly, aberrant Wnt signaling has been detected in stem cell depletion disorders and has been linked to predisposition to tumors. It is possible that these phenotypic outcomes are the result of functional and structural mitochondrial changes initiated by Wnt [62]. In a different study using HEK293 cells, overexpression of the mitochondrial protein ALEX3 (arm-containing protein lost in epithelial cancers linked to the X chromosome) generated mild to severe mitochondrial aggregation [63]. These mitochondrial phenotypes were reduced upon activation of the non-canonical Wnt/Ca^2+^ pathway, suggesting that Wnt regulates mitochondrial distribution and dynamics through ALEX3 protein degradation [64]. As ALEX3 regulates mitochondrial aggregation, dynamics and trafficking in neurons, it would be interesting to probe for interactions with Wnt modulators. The connection between ALEX3 with the Wnt pathway could be particularly relevant in maintaining mitochondrial homeostasis during neural development and maturation.

In the context of stem cells, Wnt regulation of mitochondrial function has been studied in mouse iPSCs derived from a Wolfram syndrome 2 murine model (*Cisd2*-/-), a mitochondrial-mediated disorder characterized by dysfunctional electron transport chain. CDGSH iron sulfur domain 2 (CISD2) deficiency increases Ca^2+^ levels in differentiating cells, acting as a negative regulator of the Wnt/β-catenin signaling pathway. In these iPSCs, mitochondrial number was reduced, and cristae were immature. Transcriptome analysis showed downregulation of genes related to mitochondrial localization, fission, fusion, inner membrane translocation, mitochondrial protein import, and membrane polarization and potential. When probed, canonical Wnt pathway members were significantly down-regulated. This result suggests that CISD2 may maintain the expression of these transcripts affecting the differentiation potential of these cells [65].

While it is known that the Wnt and mitochondrial dynamics pathways are important for development, the exact molecular mechanism by which they interact is not well understood. Interestingly, GSK3β has been shown to mediate the inactivating phosphorylation of DRP-1 at serine 693, decreasing its GTPase activity without affecting its inter/intramolecular interactions [66]. Moreover, the effects of Wnt signaling on mitochondrial dynamics in complex cellular architectures such as whole tissues remain elusive. For example, three-dimensional (3D) tissue structures derived from hPSCs, known as organoids, have emerged in the last few years and have proven useful for interrogating the Wnt pathway in vitro [67,68,69]. They constitute a powerful tool to examine human neural development in real time and complement previously characterized mouse phenotypes. Khacho et al., demonstrated that mouse neural stem cell self-renewal and fate decisions are influenced by downstream metabolic changes induced by mitochondrial dynamics. The disruption of this key mitochondrial feature is involved in the eventual aging and depletion of the stem cell pool in the mouse brain [39]. It would be interesting to investigate if the potential crosstalk between the Wnt and mitochondrial dynamics pathways have implications for brain development and maturation. For answering these questions, stem cells represent an advantageous model, as mitochondrial changes can be tracked throughout the course of differentiation and development.

### 2.2. Metabolic Regulation

Stem cells utilize glycolysis as a primary source of energy, converting glucose to lactate, bypassing the tricarboxylic acid (TCA) cycle at the mitochondria. The metabolic switch to glycolysis is necessary for reprogramming as mature, differentiated cells rely mostly on the TCA cycle for energy production [70,71]. Further, inhibition of glycolysis in human embryonic stem cells has been shown to result in apoptosis and cell cycle arrest [43]. These findings demonstrate that the metabolic profile of PSCs is required for pluripotent maintenance.

Dysregulation of canonical Wnt signaling plays a role in the progression of endocrine diseases such as metabolic syndrome and diabetes mellitus [72]. Studies in the adult murine pancreas demonstrate that deletion of β-catenin leads to glucose intolerance and reduced protection from high-fat-diet-induced obesity and insulin resistance [73]. The Wnt signaling pathway has also been implicated in the dysregulation of glucose homeostasis and adipogenesis during obesity. These findings demonstrate that Wnt impacts the regulation of whole-body metabolism by altering the growth rate, energy consumption, and function of different cell types at the tissue level [72].

The effects of Wnt signaling in modulating cellular energetics appear to be cell and tissue specific. In fibroblasts, canonical WNT3A/β-catenin signaling enhances mitochondrial biogenesis and O_2_ consumption [74]. In contrast, WNT3A inhibition by the repressor secreted FZD-related protein 5 (SFRP-5) reduces oxidative metabolism in adipocytes [75]. Also, when WNT3A is blocked in a non-canonical manner, increased glycolytic activity can be detected during osteoblast differentiation [76].

Cancer cells, like ESCs, rely on aerobic glycolysis, allowing for rapid generation of metabolites (e.g., nicotinamide adenine dinucleotide phosphate (NADPH) and acetyl-coenzyme A (acetyl-CoA)) to support proliferation and survival [77]. Metabolic stress has been implicated in the acquisition of stem cell-like characteristics in cancer cells through the activation of WNT3A pathway [78], but whether this feature is a reflection of a primordial embryonic state is not clear. Recent evidence indicates that interactions between Wnt and metabolic pathways mediate aerobic glycolysis [72,76]. Several studies have focused on pharmacological modulation of elements of the Wnt signaling pathway to disrupt glycolysis in cancer cells. A large number of metabolic genes (including enzymes involved in glutamine and fatty acid metabolism) were found to be β-catenin/TCF transcriptional targets in ovarian cancer [79]. Interestingly, in different breast cancer cell lines, canonical Wnt/β-catenin signaling inhibits mitochondrial respiration by down-regulation of cytochrome *c* oxidase, cytochrome *c*1, and ATP synthase subunit γ, resulting in increased aerobic glycolysis [80,81]. Non-canonical Wnt pathways have also been shown to play important roles in cancer cell metabolic reprogramming through crosstalk with the protein kinase B/mechanistic target of rapamycin (AKT/mTOR) pathway. In murine hyperplastic mammary tissue, WNT1 overexpression induces mTOR signaling by inhibiting GSK3β [82]. In prostate cancer, Wnt co-receptor LRP6 increases aerobic glycolysis in a β-catenin independent fashion by directly activating AKT/mTOR signaling [83]. In melanoma WNT5A can act through Wnt/Ca^2+^ signaling pathway generating an increase in aerobic glycolysis [84]. Further interrogation of the connections between Wnt signaling and metabolism have the potential to expand our knowledge of basic cancer mechanisms. These basic mechanisms may emerge as novel therapeutic targets for various cancer types.

## 3. Wnt-Mediated Regulation of Apoptosis

**Figure 3:**
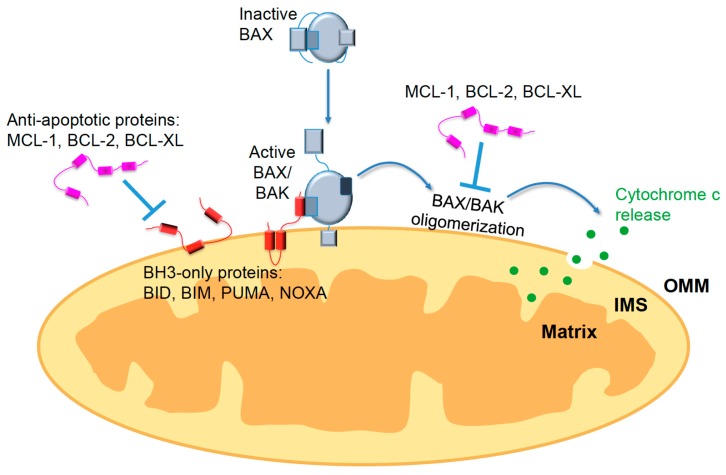
The BCL-2 family of proteins regulate mitochondrial-mediated apoptosis. After apoptotic stimuli, BH3-only proteins activate BCL-2-associated X protein (BAX) and Bcl-2 homologous antagonist/killer (BAK), which undergo a conformational change and insert into the mitochondrial outer membrane (OMM). BAK/BAX oligomerize and form pores, releasing cytochrome c from the intermembrane space (IMS) into the cytosol. When cells are not undergoing apoptosis, the anti-apoptotic BCL-2 members prevent mitochondrial outer membrane permeabilization (MOMP) by sequestering the BH3-only proteins or by inhibiting BAK/BAX oligomerization (figure modified from Walensky L. and Gaviathotis E., 2011). BCL-XL: B-cell lymphoma extra-large; MCL-1: myeloid cell leukemia 1; BID: BH3-interacting domain death agonist; BIM: BCL-2 interacting mediator of cell death; PUMA: p53-upregulated modulator of apoptosis

Proper regulation of apoptosis is critical for both development and tissue homeostasis. The B-cell lymphoma 2 (BCL-2) family of proteins modulate the intrinsic apoptosis pathway, also known as the mitochondrial-mediated pathway. These proteins interact with one another to control mitochondrial outer membrane permeabilization (MOMP) [85] (Figure 3). BCL-2 proteins assemble into similar globular structures and are characterized by multiple domains, known as BCL-2 homology domains (BH1, BH2, BH3, and BH4). The multi-domain family members can be classified as either pro-apoptotic (e.g., BCL-2-associated X protein (BAX) and Bcl-2 homologous antagonist/killer (BAK)) or anti-apoptotic (e.g., BCL-2, B-cell lymphoma extra-large (BCL-xL), Myeloid cell leukemia 1 (MCL-1)). The activating pro-apoptotic proteins, known as the BH3-only proteins, are made up of only one BH domain, the BH3 domain (e.g., p53-upregulated modulator of apoptosis (PUMA), Noxa, BCL-2 interacting mediator of cell death (BIM), BH3-interacting domain death agonist (BID), and BCL-2 agonist of cell death (BAD)). Mitochondrial outer membrane permeabilization is triggered after the BH3-only proteins activate the effector proteins BAK and BAX [86]. While BAK is mainly localized to the outer mitochondrial membrane (OMM), BAX translocates from the cytosol and undergoes a conformational change to insert into the OMM via its C-terminal domain [87]. BAK/BAX then oligomerize to form pores, releasing cytochrome *c* from the intermembrane space (IMS) [88]. This results in the assembly of the apoptosome, composed of apoptotic protease activating factor 1 (APAF-1) and caspase-9. Following this, several caspases are cleaved and activated, ultimately leading to the destruction of necessary proteins [89]. Organelles undergo condensation, the mitochondrial network fragments and collapses, and the plasma membrane encases cellular content. Apoptotic regulation, like Wnt modulation, is both context dependent and variable between cell types. Here, we will discuss potential points of convergence between these pathways in the context of PSCs.

### 3.1. Regulation of Apoptosis in Pluripotent Stem Cells

Human pluripotent stem cells undergo very rapid apoptosis after DNA damage in a p53-dependent manner [90,91]. BAX was shown to be partially responsible for this primed state, since in untreated cells that were not undergoing apoptosis, BAX was in an already active state and localized at the Golgi [90,92]. Prior to this study, active BAX was thought to be at the mitochondria exclusively in cells undergoing apoptosis. Interestingly, BAX was no longer active or localized to the Golgi after just two days of differentiation into embryoid bodies (EBs). Additionally, the levels of pro-versus anti-apoptotic proteins are shifted in hESCs (e.g., high levels of PUMA and low levels of BCL-2) to push them closer to the threshold of apoptotic initiation [91]. Increased apoptotic sensitivity in PSCs is advantageous, as they can very rapidly initiate the cell death program preventing the accumulation of deleterious mutations. Convergence between the intrinsic apoptotic pathway and Wnt could be a contributing factor to the variability observed in apoptotic sensitivity and regulation in PSCs and differentiated cells.

### 3.2. The Convergence of the Wnt and Apoptotic Pathways

The canonical and non-canonical Wnt pathways induce the expression of several genes that regulate mitochondrial apoptosis. The Wnt/β-catenin pathway modulates apoptotic potential through one of its main target genes, *Myc*. A series of studies have implicated MYC in modulating apoptotic potential. These studies served as the foundation to understand how one of the most potent pro-proliferative genes is also among the most potent inducers of cell death [93,94]. The ability of MYC and other oncoprotein transcription factors (e.g., E1A and E2F) to induce cell death in non-transformed cells has been the focus of several studies [95,96,97]. A recent study showed that MYC-deficient cells are unable to upregulate p53 due to increased levels of the E3 ubiquitin ligase MDM2 (mouse double minute 2 homolog) in intestinal, splenic, and thymic cells.

MYC plays a critical role in maintenance of mESC pluripotency and self-renewal, as well as induction of pluripotency [98,99]. MYC maintains and induces pluripotency at least in part by repressing differentiation-associated gene expression, but the molecular mechanisms of *Myc* repression of differentiation genes in stem cells are yet to be fully defined. Wnt-induced expression of MYC may promote the inherent apoptotic sensitivity of stem cells, yet this possibility remains unexplored. Additionally, elevated expression and activity of MYC is a nearly universal hallmark of human cancer [100,101,102,103]. Considering the decreased apoptotic sensitivity of cancer cells, it will be interesting to explore the interplay between canonical Wnt signaling and mitochondrial apoptosis. Moreover, as with mitochondrial dynamics, much of what we know about how MYC drives apoptosis and tumorigenesis comes from in vitro studies carried out in cell-culture systems that lack the 3D organization typical of tissues and organs.

There is also evidence that the non-canonical Wnt/DVL-JNK signaling pathway modulates apoptosis. Mouse embryonic fibroblasts (MEFs) derived from JNK1 and JNK2 knockout mice show decreased sensitivity to apoptosis [104,105]. Similarly, JNK3 knockout caused resistance to glutamate-induced cell death. This implies that JNK3 is partially responsible for activation of apoptosis in hippocampal neurons [106]. Mechanistically, JNK signaling is known to directly modulate several members of the BCL-2 family. For instance, BAX translocation to the outer mitochondrial membrane is mediated in part by JNK-induced phosphorylation of BAX anchor proteins (i.e., 14-3-3 proteins) and cytosolic BAD. BAX can then be activated and directed to the mitochondria by BAD to facilitate cytochrome *c* release [107]. Other BH3-only proteins can also be modulated by JNK activity. In particular, JNK phosphorylation of BIM and BCL-2 modifying factor (BMF) results in their release from dynein and myosin motor complexes to activate BAK and/or BAX [108]. JNK can also interact with BCL-2 to suppress its anti-apoptotic function [109]. Further investigation is needed to determine whether this modulation of the BCL-2 family and apoptosis is due to upstream signaling by Wnt and DVL-mediated JNK activation.

### 3.3. Wnt Signaling and Apoptosis in Adult Stem Cells

The Wnt pathway is a dominant factor contributing to self-renewal in many adult stem cell types, including hematopoietic stem cells (HSCs). Some studies have explored the effects of Wnt pathway modulation on the proliferation capacity of the HSC pool [110,111,112,113,114]. In an early study, overexpression of constitutively activated β-catenin in HSCs resulted in increased proliferation capacity and efficient maintenance of the HSC pool [110]. Later attempts to modulate Wnt signaling revealed that conditional overexpression of β-catenin blocked multilineage differentiation and exhausted the HSC pool, ultimately leading to lethality in mice [111,112]. This depletion was postulated to be a result of Wnt-mediated regulation of apoptosis. Supporting this idea, Ming et al., showed that overexpression of anti-apoptotic BCL-2 in HSCs could repress mitochondrial-mediated apoptosis. Even though these cells expressed active β-catenin, they were able to maintain their self-renewal capacity [113]. This phenomenon has been a topic of debate in the field, however, due to the challenges of delineating between inhibited proliferation and bona fide initiation of apoptosis, both in vitro and in vivo.

Famili et al., [114] also showed that activated Wnt signaling reduces reconstitution capacity in HSCs, but they did not observe changes in apoptosis levels. Instead, they attribute the depletion of the HSC pool to loss of stemness. In this study, WNT levels were modulated by generating several APC mutants in HSCs [114]. When compared to wild-type HSCs, the APC mutants displayed high Wnt target gene levels, reduced proliferation, and a greater tendency to differentiate. However, in contrast to the findings highlighted in Ming et al., no differentially regulated signatures of pro- or anti-apoptotic gene expression were observed [114]. Additionally, high levels of Wnt signaling induced by APC knockout did not affect apoptosis in comparison to wild-type HSCs, as shown by annexin V/7-amino-actinomycin (7-AAD) and cleaved-caspase 3 staining. While these results suggest that there is no effect of Wnt signaling on apoptosis initiation in HSCs, it would be interesting to observe these cells in response to stress. Perhaps constitutive activation of low levels of Wnt signal in HSCs could protect cells from apoptosis by promoting their differentiation or inducing the activity of non-canonical Wnt pathways.

It is clear that the role of Wnt in apoptotic regulation remains controversial. The differences in downstream effects of Wnt activation likely depend on the dosage of Wnt signals and the dependence of HSCs on canonical versus non-canonical Wnt pathways. This dependence, in turn, could attribute to variability in HSC sensitivity to apoptosis. Applying the findings from adult stem cells to PSCs from both mouse and human systems would be an exciting future direction.

## 4. Wnt and Cell Cycle

Proper function and regulation of the cell cycle is essential for normal cell growth, division, and differentiation. The cell cycle consists of gap phases separating the S phase, where chromosomes are replicated, and the M phase, where chromosomes are separated, and two identical daughter cells are produced by cytokinesis. The overall length of the PSC cycle is shorter than that of somatic cells, and the gap phases, particularly G1, are abbreviated [115,116]. However, the underlying link between cell cycle control, self-renewal, and differentiation potential in PSCs remains an active area of research. Despite its known roles in cell cycle regulation of somatic cells, the involvement of Wnt signaling in the shortened PSC cycle remains poorly characterized. Here, we will describe the intrinsic characteristics of the PSC cycle, its involvement in cellular differentiation, and potential connections to Wnt signaling.

### 4.1. The Pluripotent Stem Cell Cycle

Pluripotent stem cells have a drastically shortened cell cycle regulated by a unique set of control mechanisms. This abbreviated cycle is critical to the early development of the rapidly expanding embryo. In fact, gap phases are completely absent in the very early stages of embryogenesis. For example, in the early cells of the *Xenopus* embryo, the cell cycle is as short as 30 min [117]. Somatic cells generally have a cell cycle duration of about 24 hours, with a majority of the time spent in G1 phase, which lasts about 11 hours [118]. However, ESCs derived from the ICM of the developing human embryo have a cell cycle of about 16 hours, with a mere three-hour G1 phase [115,119,120]. Early research using mESCs determined that many of the well-characterized cell cycle control mechanisms (reviewed in [121,122]) regulating the somatic cell cycle were modified or absent in mESCs [119,123].

The most significant cell cycle control mechanism absent in mESCs is the well-characterized restriction point present at the end of the G1 phase [124,125,126]. This checkpoint serves to ensure that both the intracellular and extracellular environment are favorable for cell cycle progression, and ultimately the survival of resulting cell progeny. If either environment is unfavorable the cells will not continue to S phase, preventing DNA replication and division. The restriction point is regulated by several key proteins. A primary regulator of the restriction point is the G1/S cyclin dependent kinase 2 (CDK2), which promotes cell cycle progression through phosphorylation of key substrates in a cell cycle dependent manner [122]. While CDK2 activity normally oscillates as the cell cycle progresses, it is constitutively activated in the mESC cycle [127,128]. Additionally, CDK2 can generally be inhibited by CDK inhibitors, however, these are not expressed in mESCs [119,129]. One of the key substrates of CDK2 is the retinoblastoma (RB) tumor suppressor protein which the gatekeeper of the restriction point. RB is constitutively phosphorylated and therefore inactivated in mESCs, ultimately rendering the restriction point non-functional [127,128,129]. In hPSCs CDK activity appears to be cyclical and CDK inhibitors are expressed, albeit at lower levels than observed in somatic cells. Due to this, RB is not constitutively phosphorylated, resulting in a functional restriction point [119,120,123,130,131,132].

### 4.2. The Pluripotent Stem Cells Cycle and Differentiation

The abbreviated duration of the pluripotent cell cycle is due to the drastically shortened length of the G1 phase. Accumulating evidence indicates that G1 duration is critical to a cell’s decision to self-renew or differentiate [119,132,133,134]. For example, lengthening of G1 phase in neural stem cells (NSCs) is necessary for their differentiation into neurons [135,136,137,138,139]. In the ventricular zone of the developing mouse cortex, cell cycle duration increases from 8 to 18 hours [140]. This drastic increase in cell cycle length is the result of rapid expansion of the G1 phase, which lengthens from 3 to 13 hours [135,140,141]. Seminal studies indicate that cell differentiation decisions are initiated in the G1 phase. Research utilizing the fluorescent ubiquitination cell cycle indicator (FUCCI) system [142] determined that stem cell fate was dependent on when a differentiation signal was received during the cell cycle and that PSCs are most susceptible to differentiation signals specifically during G1 [131,138,143]. Paulkin and Vallier [143] determined that if a signal for differentiation is received early in G1, the cell differentiates towards the mesoendoderm lineage. However, if a differentiation signal is received late in G1, the cell differentiates towards the ectoderm lineage. Another interesting study demonstrated that mPSCs and mouse NSCs with a lengthened G1 phase were more prone to divide asymmetrically in differentiating conditions, while those with an abbreviated G1 underwent symmetric divisions [138]. These landmark studies not only characterized the importance of cell cycle in cell fate decisions, but demonstrated that manipulation of G1 length itself could promote differentiation.

### 4.3. A Potential Role for Wnt in G1 Phase Regulation of PSCs

The canonical Wnt/β-catenin signaling pathway has been extensively characterized as a primary driver of cell growth and proliferation [144,145,146]. During G1, Wnt signaling induces the transition to the S-phase by active transcription of *c-Myc*, promoting passage through the restriction point [145,147]. c-MYC upregulates expression of cyclin D1 and represses expression of CDK4 inhibitors p21 and p27 [145,148]. Cyclin D1 binds to and activates CDK4, which phosphorylates and inactivates RB, promoting passage through the restriction point. In this way, Wnt promotes the G1-S phase transition. However, considering the absent or decreased expression of CDK inhibitors in PSCs, as well as the differences in CDK activity, it seems unlikely that Wnt-mediated cell cycle control in PSCs would function as it does in a somatic cell.

In contrast to somatic cells, active Wnt signaling in mESCs appears to inhibit proliferation and the G1 to S transition [142]. These results seem counterintuitive, as previous studies have demonstrated that Wnt signaling is required for maintenance of self-renewal and pluripotency. Jaime-Soguero et al., determined that the Wnt mediated transcription cell factor TCF1 promotes transcription of key pluripotency factors (octamer-binding transcription factor 4 (OCT4), NANOG, and SRY-Box 2 (SOX2)). Additionally, TCF1 induced expression of inhibitors of CDK (INK4) family members following treatment with a GSK3 inhibitor [149]. INK4 proteins specifically inhibit G1 phase CDKs, including CDK4. These data suggest that active Wnt signaling inhibits the G1 to S phase transition in mESCs in contrast to somatic cells. Indeed, activated Wnt signaling increased the proportion of cells in G1 phase and decreased the number of S phase cells. However, this did not promote differentiation or decrease expression of pluripotency markers.

It is surprising that increased expression of CDK inhibitors and lengthened G1 phase did not promote differentiation. Perhaps this was due to the counteracting effect of increased transcription of pluripotency markers by TCF1. Wnt-activated transcription by TCF3 has been shown to promote mesoendoderm differentiation of both hPSCs and mPSCs [150]. However, TCF3 was not shown to be recruited to the INK4 promoter [149]. Investigation of the connection between Wnt mediated control of the G1-S phase transition is needed in hPSCs, as cell cycle control mechanisms have been demonstrated to be variable between hPSCs and mPSCs.

Variability in cell cycle control and Wnt signaling may be due to differences between the pluripotent state of mPSCs and hPSCs. As described previously, human PSCs are more similar to mouse epiblast stem cells (found in post-implantation embryos) and are considered to be primed for differentiation [27,28]. However, mPSCs are naïve. Indeed, the role and levels of Wnt activity varies greatly between the naïve and primed pluripotent states. In fact, inhibition of Wnt/β-catenin signaling in naïve PSCs can induce a more primed state [151,152]. The connection between Wnt/β-catenin signaling, regulation of the G1-S phase transition, and PSC maintenance remains unclear. It will be exciting to investigate Wnt-mediated cell cycle control mechanisms in primed versus naïve hPSCs using newly developed culture techniques [153].

### 4.4. Mitotic Regulation, Wnt, and Pluripotent Stem Cells Differentiation May Be Linked

Emerging evidence indicates that other phases of the cell cycle, particularly the G2/M phase, may also play a critical role in PSC differentiation. Recent studies demonstrate that OCT4 plays a non-transcriptional role in the regulation of the G2/M phase [154]. OCT4 complexes with CDK1/Cyclin B, ultimately inhibiting its function. Depletion of OCT4 resulted in an abbreviated G2 phase and early mitotic entry. Premature mitotic entry led to loss of chromosome integrity due to mis-segregation and ultimately apoptosis. Surprisingly, this role for OCT4 in regulation of the G2/M phase was independent of its characterized transcriptional role in PSCs. Additionally, mesodermal differentiation requires a unique G2/M pause mediated by mitotically active CDK1 [155,156]. Even in multipotent progenitors, prolonged mitotic duration has been demonstrated to promote differentiation [157]. A recent study implicates Wnt in a potential role in mitotic regulation of mESCs. Inhibition of autocrine Wnt signaling in mESCs resulted in mitotic defects and chromosome instability [158]. This phenotype led to impaired differentiation both in vivo and in vitro. However, the underlying mechanisms linking PSC differentiation potential and mitotic regulation remain elusive.

Wnt/β-catenin signaling activity peaks during the G2/M phase [159,160,161]. This increased activity directly correlates with enrichment of canonical mitotic cyclins A and B. In *Drosophila* and *Xenopus* cells, cyclin Y was recently characterized as a mitotic cyclin that complexes with the CDK-like protein CDK14, activating it during G2/M phase [159,162,163,164]. CDK14/Cyclin Y activates LRP6 by phosphorylation at key residues during G2/M, promoting release of β-catenin by Groucho, and induction of Wnt-mediated gene transcription [159,165]. Aside from its canonical function, nuclear β-catenin and several other Wnt signaling components (AXIN1, AXIN2/conductin, and APC) all localize to centrosomes, and appear to regulate mitotic spindle integrity [166,167,168,169,170,171,172]. Previous studies demonstrated that ablation of autocrine Wnt signaling in mPSCs results in significant mitotic defects [158]. Based on this, it would be interesting to investigate a potential role for Wnt-mediated regulation of the mitotic spindle in mPSCs and hPSCs and their effect on self-renewal and pluripotency. This mechanism may reveal an underlying link between the Wnt pathway and stemness, as LRP6 has been previously implicated as a key regulator of stem cell maintenance [173,174].

Additionally, Wnt-mediated regulation of mitosis plays an important role in control of asymmetric division of progenitor cells [175,176]. Asymmetric divisions occur when a progenitor cell produces both a progenitor and differentiated progeny. Symmetric divisions result in production of two identical progeny. Development of tissues is dependent upon a delicate balance between maintenance of a progenitor pool and production of differentiated progeny, so tight control of symmetric and asymmetric divisions is critical [75,177,178]. Strict regulation of cortical progenitor asymmetric division is essential for normal cortical development [157,179,180]. Increased levels of differentiating symmetric divisions in the developing cerebral cortex can lead to irregular brain size and diseases such as autism spectrum disorder (ASD) [181,182,183,184].

Polarity plays a significant role in the regulation of symmetric and asymmetric divisions [176,185,186,187]. For example, the position of a cortical progenitor relative to the apical polar membrane in the cortex affects whether the cell undergoes symmetric or asymmetric division [180,188,189]. Accumulating evidence also indicates that mitotic spindle size and symmetry plays a critical role in regulation of asymmetric divisions [145]. A recent study demonstrated that cortical progenitors exhibited spindle-size asymmetry, or unequal spindle volume on either side of the chromosomes, in developing mice [190]. Interestingly, Delaunay et al., [190] determined that cells with more spindle-size asymmetry underwent more neurogenic asymmetric divisions, producing a greater number of differentiated neurons early in development. Further investigation revealed that this effect was mediated by several components of the Wnt/PCP-signaling pathway, including WNT7A and Van Gogh-like (VANGL) planar cell polarity protein 2 (VANGL2). In fact, depletion of VANGL2 in vivo increased spindle asymmetry in the progenitor population and promoted premature cell cycle exit of these progenitors. This cell cycle exit prevented later production of cortical neurons, indicative of aberrant brain development. It will be exciting to explore a potential role for Wnt-mediated regulation of mitosis and the mitotic spindle in asymmetric division, not only in neural progenitors, but also in PSCs.

## 5. Concluding Remarks and Future Perspectives

**Figure 4:**
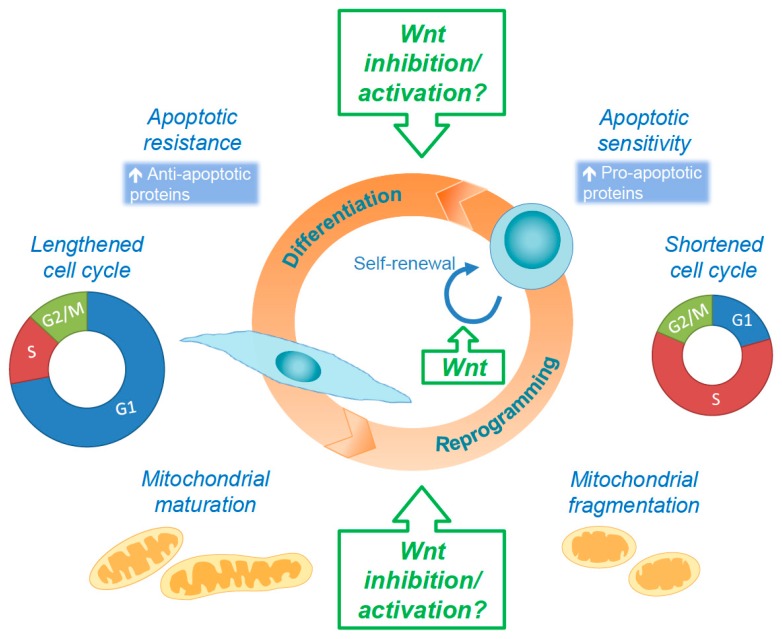
Stem cell properties of self-renewal and pluripotency and their modulation by Wnt. Pluripotent stem cells have (1) high mitochondrial fragmentation, (2) increased sensitivity to apoptosis, and (3) shortened cell cycle due to abbreviated G1 phase. These properties change dynamically over the course of differentiation. Wnt signaling pathways, both canonical and non-canonical, impacts these processes, and inhibition or activation of Wnt can promote pluripotency or differentiation.

In the past decade, there has been a clear expansion in our understanding of the regulatory circuitry and the complexity of the Wnt signaling pathway. While additional connections between the Wnt pathway and stem cell self-renewal programs have been identified, the exact molecular mechanisms underlying Wnt signaling in physiological and pathological conditions remain incompletely understood. Recent studies have revealed potential associations between the Wnt pathway, mitochondrial dynamics, apoptosis, and cell cycle, which in turn affect self-renewal and differentiation of PSCs (Figure 4).

Emerging studies demonstrate clear intersections between the cell cycle and mitochondrial dynamics pathways. As Wnt signaling activity oscillates with cell cycle progression, the mitochondrial dynamic state is variable in each phase. Mitochondria form large networks during the G1 phase and then hyper-fuse during the S phase. In the G2/M phases, mitochondria become highly fragmented [191]. Regulation of these changes in mitochondrial morphology is largely the result of CDK mediated regulation. DRP1 is activated by CDK1-mediated phosphorylation during the M phase, promoting mitochondrial fragmentation [192,193]. Additionally, RB has been demonstrated to promote the transcription of mitochondrial regulatory genes [194,195,196], further connecting cell cycle progression and mitochondrial morphology. Cell cycle regulators appear to additionally control mitochondrial function. For example, CDK1 phosphorylates complex I of the electron transport chain [197]. These studies open the intriguing possibility that Wnt signaling could play a role in regulating mitochondrial dynamics in a cell cycle–dependent manner.

The effects of Wnt signaling on self-renewal and differentiation vary between naïve and primed PSCs [23,152,198]. Understanding the requirements of Wnt signaling regulation for induction and maintenance of the naïve pluripotent state for human stem cells will facilitate their application for studying early embryonic development, disease modeling, drug screening, and cell-based therapies.

With rapid technological advances in genomics and systems biology in recent years, we remain hopeful that the connections between Wnt signaling, apoptosis, mitochondrial dynamics, and cell cycle will be strengthened by future studies. For instance, the role of Wnt signaling in the regulation of apoptosis sensitivity and the differential effects on pluripotent stem cells and differentiated cells is an area that could be explored [90,199]. Additionally, research has begun to reveal that, as stem cells exit the pluripotent state, changes in the intrinsic regulation of mitochondrial dynamics affect the ultimate fate of the cell [39,200]. It will be exciting to explore the importance of Wnt on these changes throughout development and between different cell types. Advances in our understanding of Wnt regulation will be crucial for managing various diseases in which regulatory aspects of the pathway go awry. Elucidating the contribution of the canonical and non-canonical Wnt signaling pathway to stem cell homeostasis, proliferation, and differentiation could also impact the developing areas of stem cell-based therapies and tissue regeneration.
